# New Insights into *Bacillus*-Primed Plant Responses to a Necrotrophic Pathogen Derived from the Tomato-*Botrytis* Pathosystem

**DOI:** 10.3390/microorganisms10081547

**Published:** 2022-07-30

**Authors:** Paloma Morales, Máximo González, Ricardo Salvatierra-Martínez, Michael Araya, Enrique Ostria-Gallardo, Alexandra Stoll

**Affiliations:** 1 Nees Institute for Plant Biodiversity, Bonn University, 53115 Bonn, Germany; pmorales@uni-bonn.de; 2 Centro de Estudios Avanzados en Zonas Áridas (CEAZA), La Serena 1720256, Chile; rsmesteban@gmail.com (R.S.-M.); enrique.ostria@ceaza.cl (E.O.-G.); 3 Instituto de Investigación Multidisciplinar en Ciencia y Tecnología, Universidad de la Serena, La Serena 1720170, Chile; 4 Centro de Investigación y Desarrollo Tecnológico en Algas CIDTA, Universidad Católica del Norte, Coquimbo 1781421, Chile; mmaraya@ucn.cl

**Keywords:** ISR, priming, resistance induction, biocontrol, PGPR

## Abstract

Induced systemic resistance (ISR) is one of the most studied mechanisms of plant–microbe interaction and is considered a very promising alternative for integrated pest management programs. In our study, we explored the plant defense response induced by *Bacillus velezensis* BBC047 in relation to its application before or after *Botrytis cinerea* infection of tomato plants. The inoculation of BBC047 did not considerably alter the gene expression of the tomato tissues, whereas infection with *B. cinerea* in BBC047-primed plants induced expression of LRR and NBS-LRR receptors, which are highly related to the ISR response. As expected, *B. cinerea* infection generated molecular patterns typical of a defense response to pathogen infection as the overexpression of pathogenesis-related proteins (PRs) in leaflets distant to the point of infection. The curative treatment (P + F + B) allowed us to gain insights into plant response to an inverted priming. In this treatment, *B. cinerea* caused the m tissue damage, extending nearly entirely across the entire infected leaves. Additionally, genes generally associated with early SAR response (<16 h) were overexpressed, and apparently, the beneficial strain was not perceived as such. Therefore, we infer that the plant defense to the curative treatment represents a higher degree of biological stress triggered by the incorporation of strain BBC047 as second arriving microorganism. We highlight the importance the phytosanitary status of plants prior to inoculation of beneficial microorganism for the biocontrol of pathogens.

## 1. Introduction

Tomato (*Solanum lycopersicum*) is one of the most economically important crops in the world, with a production of nearly 187 million t in 2020 harvested from 6.1 Mha [1]. Moreover, in plant science, the tomato is one of the most studied crops, particularly its genetics, cellular processes and molecular biology [2]. Despite these efforts, the low genetic diversity in domesticated tomatoes leads to high susceptibility to pathogens; more than 200 diseases can cause significant losses during cultivation and after harvest [3,4]. Among all phytopathogenic fungi that affect tomato crops, *Botrytis cinerea* is the second most prevalent cause of economic losses worldwide [5]. No resistant tomato cultivars have been reported to date. Therefore, synthetic pesticides are mainly used for *B. cinerea* control, which increases fungal resistance [6].

At present, multiple management strategies are available to replace or reduce the application of synthetic fungicides in agriculture, highlighting the use of plant-growth-promoting rhizobacteria (PGPR) as biocontrol agents [7,8]. *Bacillus* strains have been shown to be efficient in controlling *B. cinerea* through direct and indirect mechanisms [9,10]. Direct antagonism relies on the excretion of secondary metabolites and their antibiotic and/or inhibitory effect on the pathogen. The most studied indirect mechanism employed by *Bacillus* strains resembles the plant defense mechanism and enhances their resistance to pathogen infection [11]. In this context, the lipopeptide surfactin, produced by multiple *Bacillus* strains, is recognized as an elicitor by the plant [12,13], and its production can be modulated by root exudates [14]. Surfactin is also a key molecule for the assembly of *Bacillus* biofilms and plant colonization [15,16,17].

Plant defense mechanisms against pathogens constitute a very complex system, wherein pathogen perception plays a central role [18]. In this context, at least two types of response can be differentiated: microbe-associated molecular pattern-triggered immunity (related to systemic acquired resistance (SAR) initiated by a localized pathogen infection) and microbial effector-triggered immunity (related to induced systemic resistance (ISR)) [11,19]. In both cases, plant defenses are preconditioned by prior contact with a pathogenic or beneficial microorganism, respectively, showing an efficient response to pathogen infection in distal parts of the plant far from the site of exposure to the elicitor. Despite the exact purpose of both mechanisms, they differ in terms of the nature of the triggering elicitor and the controlling hormone signaling pathways. SAR is activated by a pathogen-associated molecular patterns (PAMPs), followed by an early increase in endogenous salicylic acid (SA) and the expression of pathogenesis-related genes (PR genes) [19,20]. On the other hand, ISR is triggered by beneficial microorganisms, such as *Bacillus* spp., after recognition of its associated elicitor molecules (microbial-associated molecular patterns, MAMPs) [21], which puts the plant immune system into alert status, also called priming. ISR has been mainly related to jasmonic acid (JA) and ethylene (ET) signaling, oxidative burst and the expression of disease resistance genes (R genes) belonging to a gene family encoding for nucleotide-binding site leucine-rich repeats (NBS-LRR) proteins [22]. Additionally, compared to SAR, ISR is specific in its epigenetic regulation, transcriptional and posttranscriptional changes and hormonal signaling [11].

The authors of recent studies have described ISR activation as depending on SA [20,23,24]. For example, Beris et al. [23] showed that resistance induced by *Bacillus amyloliquefaciens* MBI600 against Tomato spotted wilt virus and Potato virus Y is dependent on SA. *Bacillus cereus* AR156 induces ISR, employing signaling pathways that are simultaneously dependent on SA and JA/ET [24]. Such findings demonstrate that despite consistent research on ISR, many questions remain regarding the underlying mechanisms that allow for the development of the phenomena known as priming and resistance to necrotrophic fungi.

In this work, using *B. cinerea* as a necrotrophic fungus model, we explore the mechanisms associated with ISR induced by *B. velezensis* BBC047, a strain known to trigger ISR [17,25]. We designed an experiment to identify systemic gene responses via RNAseq analysis in tomato plants treated with strain BBC047 before and after infection with *B. cinerea*. Our results provide new insights into crucial aspects of this interaction in the context of priming and activation of induced systemic resistance (ISR).

## 2. Materials and Methods

### 2.1. Plant and Microorganism Materials

In this study, the ‘Cal Ace’ hybrid tomato cultivar (PetoSeed Co. Chile LTDA) was used as host plant. All plants were germinated and grown in a plug tray filled with peat. After one month of growth, plants with a second set of true leaf developed were taken out of the plug tray, their roots were completely cleaned with tap water and washed with sterile water and placed in a sterile nutrient solution. After three days in the sterile solution, the plants were transferred to a gnotobiotic system. To this end, the roots were sterilized by immersion in a solution of 70% ethanol for 1 min and then in a solution of sodium hypochlorite 5% for 5 min (protocol adapted from McKinnon [26]). Roots were subsequently washed five times with sterile distilled water and placed into a fresh sterile nutrient solution until a resistance induction assay was performed.

The strain *Bacillus velezensis* BBC047, previously described by Salvatierra et al. [25] and Stoll et al. [17], was used as a biocontrol agent. For inoculum production, strain BBC047 was grown in liquid Luria-Bertani (LB) medium at 30 °C at 170 rpm for 12 h until reaching a concentration of 10^8^ CFU/mL. The bacterial cells were collected and resuspended in sterile water, and the concentration was adjusted to ~10^6^ CFU/mL for inoculation of the resistance induction assay.

Finally, *Botrytis cinerea* was used as the fungal pathogen. It was maintained by subculturing every two weeks in potato dextrose agar (PDA) medium. For the resistance induction assay, plant infection was performed using two PDA disks with a diameter of 5 mm containing sporulated *B. cinerea* (11 days old), which were placed on two leaflets of each tomato plant.

### 2.2. Resistance Induction Assay and Treatment Application

**Figure 1:**
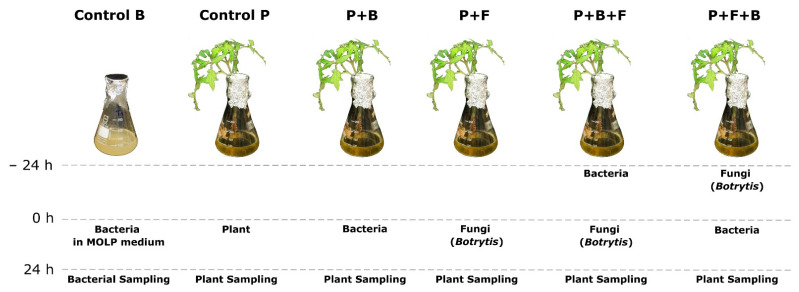
Gnotobiotic resistance induction assay with strain BBC047 in tomato plants infected with *Botrytis cinerea*. Six treatments are displayed: control bacteria (CB), control plants (CP), plant + bacterial treatment (P + B); priming treatment (P + B + F), where BBC047 was inoculated 24 h before *B. cinerea* infection; curative treatment (P + F + B), where tomato plants were infected with *B. cinerea* and, 24 h later, were inoculated with BBC047; and infection control (P + F), where tomato plants were infected with *B. cinerea* only.

The resistance induction assay comprised six treatments. The plants kept the gnotobiotic system were transferred individually to 250 mL Erlenmeyer flasks with 100 mL of optimal medium for lipopeptide production (MOLP). Then, the following treatments were applied: control bacteria (CB), BBC047 alone in MOLP medium; control plants (CP), tomato plants alone in MOLP medium; plants with bacterial treatment (P + B), BBC047 and plants placed simultaneously in the medium; priming (preventive) treatment (P + B + F), plants and BBC047 placed in the medium for 24 h; later, the plants were changed to a new MOLP medium and infected with *B. cinerea* using the protocol described above; curative treatment (P + F + B), the plants were first infected with *B. cinerea* and, 24 h later, changed to a new MOLP medium, which was inoculated with BBC047; and infection control (P + F) plants with two leaflets of each the tomato plant inoculated with *B. cinerea* (Figure 1). 

In the treatments with both microorganisms, either BBC047 (priming, P + B + F) or *B.cinerea* (curative, P + F + B) was applied 24 h before, and treatment was completed (with the application of the other microorganism) at the same time as all other treatments. The assay started at the moment when, in all treatments, the plant—bacteria—fungus pathosystem was completed (Figure S2).

After the assembly of each treatment, the flasks were placed under agitation at 120 rpm in orbital shakers (Grant Bio PSU 20i., Shepreth, UK) in a growth chamber at 25 °C for 24 h (12 h day/12 h night). Each plant corresponded to one experimental unit, with nine replicates (plants) established per treatment.

### 2.3. Evaluation of Disease Severity

At the end of the resistance induction assay, the disease severity was evaluated around the point of infection with *B. cinerea*. To this end, infected leaves were photographed with a square centimeter of graph paper as size standard. In ImageJ software [27], the analytical scale for picture was set in reference to the square centimeter standard. Later, the lesion area around the point of infection was quantified for each leave. For each treatment, 10 leaflets (5 plants) were evaluated. A disease reduction index (DR) was calculated using the following formula:

$$\mathrm{DR}\;(\%)=100\;-\;\frac{\left(x\;\times \;100\right)}{y}$$

where:

x = the average area of the lesion around the point of infection from 10 leaflets per treatment; and

y = the average area of the lesion around the point of infection from 10 leaflets in infection control (P + F).

### 2.4. Isolation of Lipopeptides and UHPLC-MS Analysis

For lipopeptide analysis, ultra-high performance liquid chromatography tandem mass spectrometry (UHPLC-MS) was used. After 24 h, the liquid bacterial broth from three individuals of each treatment was collected and pooled, and 100 mL was used for lipopeptide analyses (3 pooled replicates from three individuals each). Bacterial cells were removed by centrifugation at 10,000 rpm for 15 min. From the cell free supernatant, the cyclic lipopeptides (cLPs) were obtained by the acid precipitation method described by Alvarez et al. [28], with some modifications. Briefly, the cLPs were precipitated by the addition of 3N HCl until reaching a final pH of 2.0. The treated samples were kept at 4 °C for 30 min and centrifuged at 10,000 rpm for 15 min, and supernatant was discarded. The precipitated cLPs were recovered and resuspended in 1 mL of 100% methanol hypergrade for LC-MS and stored at −80 °C until UHPLC-MS analysis.

Twenty microliters of the methanolic fraction, was injected into a UHPLC Dionex UltiMate 3000 system coupled with an Orbitrap Q exactive focus detector (Thermo Scientific) equipped with a Hypersil gold column (50 × 2.1 mm, 1.9 μm). A gradient of acetonitrile and water acidified to 0.1% with formic acid was used, which allowed for the simultaneous detection of the three families of cLP. Elution was initiated with acetonitrile from 0% to 100% over 6 min at a flow rate of 0.40 mL/min. The acetonitrile percentage was then maintained for 5 min, and the column was stabilized with the acidified aqueous phase for 2 min. Data were processed using Xcalibur 4.0 software. cLPs were identified based on their retention times and mass fragments compared to the *Bacillus velezensis* strain FZB42 [29] and a surfactin standard (CAS N° 24730-31-2, Sigma Aldrich, Taufkirchen, Germany). Detection was carried out using electrospray ionization ((+) ESI). Measurements were recorded in full scan mode (scan range: 200–1500 *m*/*z*). For surfactin quantification, a calibration curve was generated by injecting 4 known surfactin concentrations (2.5, 25, 50 and 100 μg/mL), from which the concentration of surfactin was calculated for each sample (Figure S1).

### 2.5. RNA Isolation from Tomato Leaflets

For RNA isolation, leaflets (100 mg) of each replicate of each treatment were collected, frozen immediately in liquid nitrogen and stored at −80 °C until use. Therefore, leaflets different from those infected with *B. cinerea* were taken (Figure S2). Stored samples were ground in a mortar using liquid nitrogen; then, the material was processed using an RNeasy mini kit (Quiagen; Hilden, Germany) according to manufacturer’s specifications. Subsequently, RNA was used for quantitative PCR (qPCR) analysis and RNAseq library construction. Libraries were prepared with a TruSeq stranded mRNA-seq kit and sequenced with Illumina sequence platform HiSeq 3000 at the Greehey Children’s Cancer Research Institute (San Antonio, TX, USA).

### 2.6. RNAseq Data Processing and Differential Gene Expression Analysis

Raw data were preprocessed using Trim Galore Software Version 0.6.5. (Download from: https://www.bioinformatics.babraham.ac.uk/projects/trim_galore/) to remove low-quality data, Illumina adapter sequences and readings of less than 50 bp. The remaining reads (clean reads) with a minimum length of 50 bp and a quality score of Q > 20 were mapped to the tomato (*Solanum lycopersicum*) genome version ITAG 3.0 using TopHat version 2.1.1 [30,31]. The obtained BAM files were used to estimate the abundance of transcripts (FPKM, fragments per kilobase of transcript per million mapped reads) and to identify differentially expressed genes through the Cuffdiff module incorporated in Cufflinks software [30]. A differential expression from 4-fold change and *p* < 0.05 were considered as significant.

### 2.7. Principal Component Analysis (PCA), Self-Organizing Map (SOM) Clustering and Functional Annotation

Normalized RPKM expression values were used to cluster assembled contigs based on expression patterns [32]. To detect the effects of the interactions among the plant, bacteria and the fungus on gene expression, only differentially expressed genes were analyzed. The scaled expression values within samples were used to cluster these genes for a multidimensional 3 × 2 hexagonal SOM throughout 100 training iterations processed using the Kohonen package in R [33]. After the iteration process, the final assignment of genes to the winning units shaped the clusters of genes (hereafter termed nodes) associated with each set of combinations among plant, fungus and bacteria (see details above). In addition, for each node, the box plot option from the ggplot2 package in R was used to visualize the gene accumulation patterns associated with the treatments. Finally, those nodes that showed higher genes accumulation patterns for a given treatment were annotated for GO terms using GOATOOLS v0.8.2 software [34].

### 2.8. Differential Gene Expression Evaluation by Quantitative PCR

A total of 8 genes identified from RNAseq analysis were studied through qPCR analysis (Supplementary Table S1). To realize quantitative gene expression analysis, cDNA was synthesized from the total RNA (1 µg) of each sample using a PrimeScript RT reagent Kit with gDNA Eraser (Takara, Dalian, China) according to the manufacturer’s specifications. Reverse transcription was carried out at 37 °C for 15 min and at 85 °C for 5 s. in an XP thermal cycler block (Hangzhou Bioer Technology Co. Ltd., Hangzhou, China). Each qPCR reaction was carried out with Takyon ROX-SYBR master mix (Eurogentec, Seraing, Belgium) according to the manufacturer’s instructions using 20 pM of each selected primer. Amplifications were performed in a Stratagene Mx3000p qPCR system using the following cycles: 95 °C for 30 s followed by 40 cycles of 95 °C for 5 s.

To ensure an equal amount of RNA in each treatment, tomato actin gene was used as an internal control (housekeeping) [35]. Ct values were transformed using the 2^−ΔΔCt^ method for quantification of relative expression [36]. All qPCRs were performed in three biological replicates with three technical replicates.

## 3. Results

### 3.1. Disease Severity and Surfactin Production

**Figure 2:**
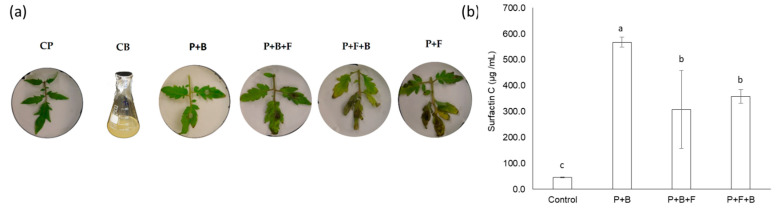
Effect of tomato root–*Bacillus* interaction on *B. cinerea* infection and surfactin C production. (**a**) Phenotype of representative leaflets per treatment infected with *B. cinerea*. (**b**) Adjusted surfactin C production in the four bacterial treatments normalized by the log of the colony-forming units (cfu) per milliliter of medium (cfu/mL) at 24 h. Different letters above the bars represent significant differences between the treatments according to the Tukey test (*p* < 0.05); *n* = 3 (three pooled replicates from three individuals each).

Disease severity was calculated as disease reduction index to identify the effects of the priming or curative application of strain BBC047 against *B. cinerea* infection in tomato plants. The priming treatment (P + B + F) showed the best performance against *B. cinerea*, reducing the lesion area by 62% compared to the other treatments. In contrast, the curative treatment (P + F + B) showed a similar necrotic area as the infection control (P + F) (Figure 2 and Figure S3).

On the other hand, the synthesis of cLP was modified due to interaction between the strain and tomato roots (Figure 2). The measured concentration of all three families of cLPs (iturins, fengicins and surfactins) was significantly reduced in the presence of plant roots (P + B, P + B + F and P + F + B) (Figure S4). Furthermore, bacterial growth was noticeably reduced when strain BBC047 was inoculated on tomato roots (Table 1).

The amount of surfactin C produced by BBC047 also varied among the plant treatments (Figure 2b, Table 1). The highest surfactin C concentration was identified in the P + B treatment, followed by CB, whereas for both B.cinerea-infected treatments P + B + F and P + F + B, significantly less surfactin C was measured. When normalizing the surfactin C concentration by the bacterial cell count (expressed as colony-forming units (cfu)/mL), the highest value was detected in P + B, although P + B + F and P + F + B still contained eight times more surfactin C/cfu than CB.

### 3.2. RNA Sequencing and Differentially Expressed Genes (DEGs) in Response to Priming and Curative Treatments

An RNAseq analysis was realized to identify the defense response genes activated in the tomato plants in interaction with the microbial treatments (strain BBC047, *B. cinerea* infection or both microorganisms in the pathosystem). An average of 109,789,542 raw reads were generated from each library, whereas a total of ~109,607,976 clean reads were obtained after removing low-quality reads and trimming the adapter sequences. Consequently, ~92.3% of the clean reads could be mapped to the tomato reference genome. Only ~0.9% of these reads showed multiple alignments, i.e., both values similar between all libraries (Supplementary Table S2). An average of 23,860 and 43,873 of known genes and their isoforms were obtained for each library, respectively. In addition, an average of 2555 and 3462 genes and isoforms corresponded to novel genes not annotated in the reference genome (Supplementary Table S3). Finally, 22,751 and 46,085 genes and isoforms were shared between the five libraries, respectively.

Significant differences in gene expression between the genes of each library were determined with a DEG test. Among control plant (CP) and C + B treatments, just 11 genes were differentially expressed after 24 h of root inoculation with strain BBC047. However, 205 genes were differentially expressed between P + F and P + F + B treatments. The highest DEGs (226) were observed between priming (P + B + F) and curative (P + F + B) treatments, revealing that different mechanisms are triggered according to the order in which the pathosystem “plant–bacteria–fungi” is ultimately assembled (Supplementary Table S4). In addition, eight differentially expressed genes were selected to validate the RNAseq assembly through qPCR analysis (Figure S5).

### 3.3. Transcript Expression Patterns of Tomato Plant across Interactions with Botrytis cinerea and/or BBC047 Strain

**Figure 3:**
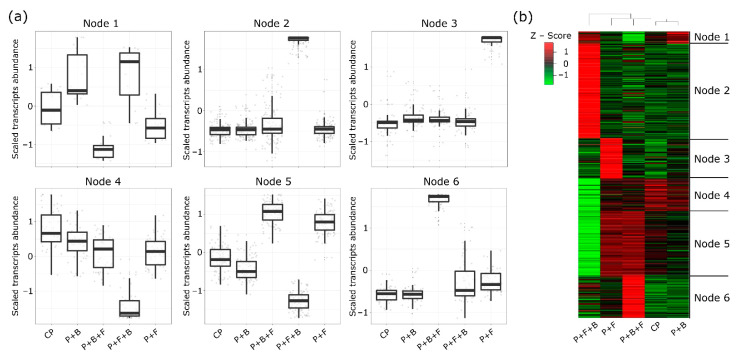
Differential gene expression (DGE) analysis by self-organizing map (SOM) and heat map. (**a**) SOM analysis with six nodes representing unique gene expression patterns in each treatment. In the box plots, lines and horizontal bars represent median, minimum and maximum of gene expression values, respectively. (**b**) Heat map developed from DGE analysis of gene expression of the four treatments, including SOM clustering identification.

Transcripts with similar patterns of accumulation were identified through self-organized map (SOM) clustering in order to determine the transcriptional dynamics of tomato plants during the resistance induction assay (Supplementary Table S5). SOM analysis showed clusters (nodes) exhibiting a specific profile for several treatments: infection control P + F (Node 3), priming treatment P + B + F (Node N6) and curative treatment P + F + B (N2 and N4) (Figure 3a,b).

**Figure 4:**
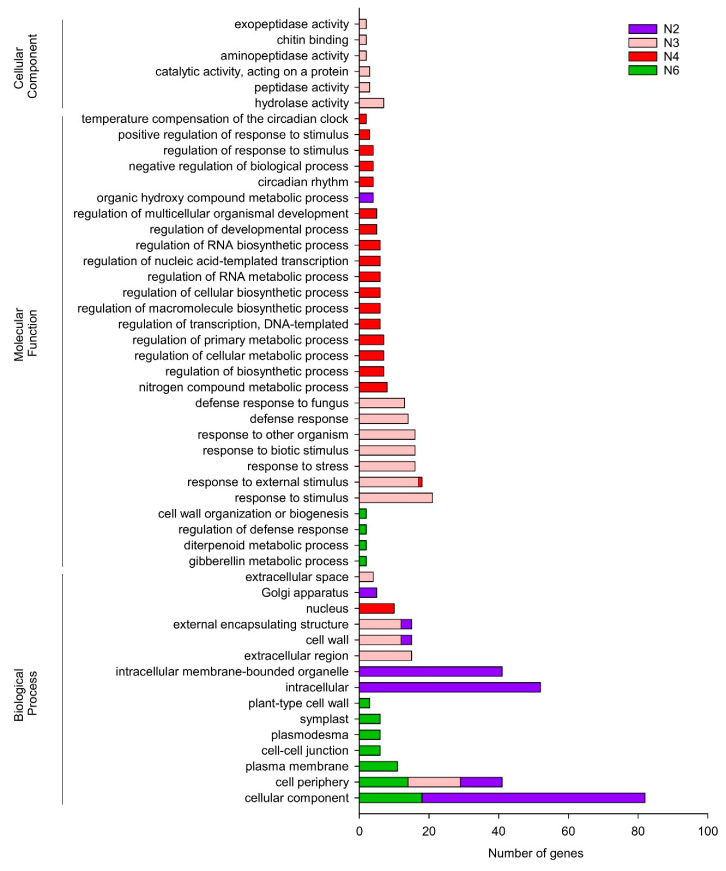
Gene ontology (GO) term enrichment analysis of differential gene expression in the four most informative nodes (N) from SOM analysis. Gene function and relative gene enrichment represent response in the plant in N2-overexpressed genes in the curative treatment (P + F + B), N4-downregulated genes in the curative treatment, N3-overexpressed genes in the infection treatment (P + F) and N6-overexpressed genes in the priming treatment (P + B + F).

To determine which processes were more affected after inoculation with BBC047, *B. cinerea* or a combination thereof, the most relevant GO terms identified in the SOM nodes were classified as biological process, molecular functions and cellular components (Figure 4).

Node 3 (N3) was associated with plant response to *B. cinerea* infection (P + F). GO analysis showed that the plant is enriched with responses associated with defense against biotic stimuli, such as fungus infection, as well as with responses to stress and development of external encapsulating structure, including cell wall, extracellular region and cell periphery (Figure 4). The following items of the cellular component category were identified: hydrolase activity, peptidase activity, aminopeptidase activity, exopeptidase activity, catalase activity and chitin-binding component (Figure 4). 

The most relevant genes associated with the previous GO classification were a group of 31 genes, all classified as pathogen-related (PR) genes (Table 2). Among the PR genes were one pathogenesis-related protein 1 (PR1), β 1,3-glucanase (PR2), one chitinase (PR3), one pathogen-induced protein (PR4), two thaumatin-like proteins (PR5), twenty-one proteinase inhibitors (PR6) and two ribonucleases (PR10). In general, all of the mentioned genes have been described as pathogenesis-related proteins (PR) (Table 2). In addition, N3 contained the upregulation of arginase 2 (Solyc01g091170.3) and acetylornithine deacetylase (Solyc08g076970.3), both genes involved in nitrate metabolism in arginine metabolism for polyamine biosynthesis (Supplementary Table S6).

On the other hand, N6 was associated with expressed genes in plants treated with strain BBC047 previous to *B. cinerea* infection and priming treatment P + B + F, which are related to the following biological process categories: cellular, component, cell periphery, plasma membrane, cell–cell junction, plasmodesma, symplast and plant-type cell wall (Figure 4). In addition, the following genes of the molecular function category were identified: genes associated with cell wall organization, regulation of defense response, diterpenoid metabolic processes and gibberellin metabolic processes (Figure 4). Specifically, N6 showed an effect of strain BBC047 in the priming treatment, which induces ISR-activated pathogen-related receptors and intracellular effector receptors involved in pattern-triggered immunity (PTI), as well effector-triggered immunity (ETI) (Table 3). Fourteen genes from this node were codified to pattern-recognition receptors (PRR), of which thirteen were annotated as transmembrane receptor-like kinase (RKL) proteins and one as a cytoplasmic nucleotide-binding leucine-rich repeat (NBS-LRR) disease protein. Of the RKL proteins, 10 were found to contain a leucine-rich motif, of which 7 were associated with an already annotated LRR protein kinase. Solyc09g083200.3.1 was annotated as Nod factor receptor protein, an RLK protein with a lysin motif (LysM) in the extracellular compartment, and its Arabidopsis homologue was aligned with the LysM-containing receptor kinase4 (LYK4). In the priming treatment, an upregulation of a key cytoplasmatic receptor was observed, displaying effector-triggered immunity (ETI) and annotated as the disease protein with nucleotide-binding motif and leucine-rich repeat domains (NBS-LRR, Solyc04g007070.3). Finally, other overexpressed genes were associated with the Arabidopsis homologous suppressor of BIR1 (SOBIR1) precursor protein, wall-associated receptor kinase 1(WAK1), suppressor of NPR-1, constitutive 4 (SNC4), flagellins-sensing 2 (FLS2) receptor and two genes homologous to leaf rust 10 disease-resistance locus receptor of Arabidopsis (LRK10L, Solyc05g008950.3.1 Solyc05g008960.3.1) (Table 3).

In addition, in N6, expression of detoxification and cell wall synthesis genes 2 glutathione S-transferase (GST), oxalate oxidase-like germin (Solyc07g041720.1) was observed, as well as the transcription factors WRKY39, WRKY 81 and NAC6 in the priming treatment. (Supplementary Table S7).

On the other hand, curative treatment P + F + B was the unique treatment with two nodes in the SOM analysis, where N4 and N2 were associated with repressed and overexpressed genes, respectively. GO analysis of N4 revealed that the primary metabolism was highly repressed (Figure 4). Interesting, in N4, a putative receptor, TIR-NBS-LRR (Solyc01g102850.2.1), and wound responsive protein (Solyc02g083310.3.1) were repressed, as well as the anthranilate synthase (first enzyme of tryptophan biosynthetic pathway) and the transcription factor TCP270 (transcription factor involved in jasmonate biosynthesis, Solyc02g094290.1). Furthermore, Caffeoyl-CoA O-methyltransferase (CCoAMT, Solyc04g063210.3), involved in lignin biosynthesis, and LEXYL2 (β-xylosidase, Solyc01g104950.3), a hemicellulose hydrolase widely found in the plant cell wall, were both repressed (Supplementary Table S8).

N2 over-represented GO terms associated with cellular components, cell periphery, intracellular and organic hydroxy compound metabolic processes (Figure 4). Three heat shock proteins were found and annotated as two HSP70 (Solyc11g066100.2, Solyc04g011440.4) and HSP90 (Solyc06g036290.3). In addition, our results showed overexpression of the transcription factors MYB75 and MYB58, which are a repressor and an activator of lignin biosynthesis, respectively; as well as the enzymes cinnamate-4-hydroxylase (C4H, Solyc06g082535.1), hydroxycinnamoyl-CoA shikimate/quinate hydroxycinnamoyl transferase (HCT, Solyc06g074710.1, 4-coumarate:CoA ligase-like protein (4CL) (Solyc) and caffeic acid O-methyltransferase (COMT, Solyc10g085830.2), all enzymes involved on lignin biosynthesis (Supplementary Table S8).

Additionally, genes associated with lipid metabolism were over-represented. Two GDSL esterases/lipases were found (Solyc03g111550.3, Solyc02g071620.3), as well as two enzymes involved in phosphatidic acid production: diacylglycerol (DAG) kinase (Solyc03g115370.3) and phospholipase PLDb2 (Solyc01g091910.4). Furthermore, two inositol-1,4,5-trisphosphate (IP3) 5-phosphatase (Solyc02g087425.1; Solyc06g054010.3), which are involved in the inactivation of IP3. On the other hand, some genes involved in cutin synthesis and deposition, as well as cell wall modification, were found—specifically, the enzyme glycerol 3-phosphate acyltransferase (GPAT, lipid synthesis and cutin deposition) and glycerol 3 phosphate transporter (Solyc03g093140.3). Additionally, ABC transporter (Solyc01g105450.3), lipid transfer protein (Solyc01g105010.3), four cytochrome P450 (Solyc03g111995.1, Solyc03g116630.3, Solyc07g055560.3 and Solyc10g083690.3) and one thioredoxin H (Solyc05g006870.3) (Supplementary Table S8) were overexpressed.

Finally, genes associated with hormonal synthesis or signaling transduction were found in N2. Component ethylene signaling pathway was identified in one ethylene receptor, ETR4 (Solyc06g053710.3), as well as two transcription factors: ERF.D4 (Solyc10g050970.1) and ERF.F5 (Solyc10g009110.1). Moreover, 9-cis-epoxycarotenoid dioxygenase2 (Solyc08g016720.1), a main enzyme involved in ABA synthesis, was over-represented, as well as two JA repressors (JAZ3 and JAZ8; Solyc07g042170.3 and Solyc08g036620.3, respectively) (Supplementary Table S8).

## 4. Discussion

### 4.1. Molecular Indicators for ISR

ISR is defined as the enhancement of the defensive mechanism in a host plant triggered by its interaction with a non-pathogenic microorganism, which is observed at the moment of the pathogen challenge [11,37]. In this work, bacterial pre-inoculation significantly reduced the necrosis area on leaves compared with the control treatment, showing an enhanced performance against pathogen infection and suggesting that bacterial pre-inoculation triggered ISR in the host plant.

Previously, Stoll et al. [17] reported that an application of surfactin treatment or BBC047 on tomato roots induced ISR. In all treatments, BBC047 strain produced the three main families of cLP, as previously published by Salvatierra et al. [25]. However, our results show that during the first 24 h of interaction with the plant roots, surfactin production (normalized by cfu) significantly increased, which is consistent with results reported by Debois et al. [14]. The amount of produced surfactin is beyond the threshold of 5 µg/mL reported to trigger a plant response in the context of ISR [12]. The ISR state was also validated by a reduction in lesion area caused by *B. cinera* in the P + B + F treatment.

To identify the molecular mechanism behind the priming treatment, RNAseq analysis was performed. The first layer of plant immune resistance mechanisms is based on pattern recognition receptors (PRRs), which detect PAMPs or damage-associated molecular patterns (DAMPs) [38]. In the priming treatment with BBC047, a specific enrichment of plant innate pattern recognition receptors (PRRs) with a kinase domain was observed, showing the activation of the defense mechanisms. PRRs are transmembrane receptors that bind to PAMPs and serve as early warning signals in the immune system.

Priming by BBC047 activates overexpression of *B. cinerea* resistance receptors. All genes described in Table 3 play a key role in sensing internal and external signals, particularly detection of the first contact between the host plant and the pathogen, in addition to transducing downstream signaling [39,40]. The LRR MIK2-like kinase reported in this work is a homologue of that reported by Coleman et al. [39], where LRR MIK2 is a crucial component of the early immune responses to a fungal elicitor.

Among the RLK genes, the annotated Nod receptor protein (Solyc09g083200.3.1) had the most significant overexpression in DEG analysis. In leguminous plants, the Nod factor receptor is part of the lysine motif (LysM) domain-containing receptor-like kinase (LYK) family of proteins, which were shown to be a receptors for the nodulation factors (NFs), modified chito-oligosaccharides produced by rhizobia and essential for establishment of nitrogen-fixing symbiosis [41,42,43,44,45]. Its *Arabidopsis* homologue, LYK4, is a plasmatic membrane receptor involved in pathogen resistance through chitin sensing signaling, induction of chitin-responsive genes and chitin-induced intracellular calcium concentrations [46]. The homology between LYK4 and our annotation suggests that the tomato gene model described in this study as Nod factor is more likely to be a pathogen receptor located in the plasma membrane related specifically to chitin recognition and associated with Ca^2+^ signaling cascade [46]. We also observed overexpression of the gene suppressor of BIR1 (SOBIR1, Solyc03g111793), a transmembrane RLK reported as a component of a tripartite complex that mediates the recognition and transduction of microbe-derived patterns, such as necrosis and ethylene-inducing peptide 1-like proteins (NPLs) [47], and it also has been shown that plants lacking SOBIR1 are susceptible to *B. cinerea* infection [48]. On the other hand, in our study, we detected receptors that not only detect PAMPs but also molecules released by the same plant, such as oligogalaturonides (OGs, related to plant cell wall), which are detected by wall-associated kinase 1 (WAK1, Solyc10g076550.1) during *B. cinerea* infection. This finding also suggests that BBC047 strain might activate cell wall damage recognition in priming treatment, preparing the plant for *B. cinerea* or other necrotrophic fungus attacks [49].

Interestingly, an NBS-LRR gene was found to be significantly overexpressed only in the priming treatment. NBS-LRR proteins are cytoplasmic receptors codified by disease-resistance genes (R genes) and key components in ETI signaling [39]. ETI is defined as the second layer of defense characterized for specific recognition of molecules secreted by the pathogen (effectors), leading to a rapid defense reaction, such as HR and prevention of pathogen spread in front of further infections. This gene might by highly related to fungal recognition, as it was aligned with the putative late blight resistance protein R1B-23 homologous gene in *S. lycopersicum*, as well as to the resistance to *Peronospora parasitica* protein 13 (RPP13) in *Arabidopsis*. Taking all this evidence together and given that only BBC047 pre-inoculation activates NBS-LRR gene expression in distal post-infection leaves and is involved adaptive defense mechanisms towards fungal infection, the presence of NBS-LRR genes might be a molecular marker of the ISR mechanism in plants, at least in the present pathosystem for fungal infection.

On the other hand, the infection control (P + F) treatment resulted in an accumulation of pathogenesis-related proteins (PR proteins) as a typical plant response to pathogen infection. The overexpression of PR genes is often related to SAR [50], specifically PR1, which is known as a molecular indicator of this mechanism [51,52,53]. Here, 31 PR genes were specifically overexpressed in the P + F treatment, whereas the PR1 gene was overexpressed significantly in both P + F and P + F + B (curative) treatments. This defense mechanism was not found in the priming treatment, showing the influence of the BBC047 strain in the plant response to *B. cinerea* infection.

### 4.2. Gene Expression in the Priming Treatment with BBC047 Strain

A hypothesis on the molecular mechanism of priming suggests that chromatin modifications prime the defense genes for faster and more robust activation [54]. In addition to receptors, the transcription factors WRKY39, WRKY 81 and NAC6 were overexpressed in the priming treatment. Transcription factors are involved in a broad spectrum of processes for interconnected pathways, of which the WRKY transcription factors are one of the most studied classes. In this work, WRKY39 had one of the most significant upregulations in the priming treatment, which might play a key role in the ISR triggered by BBC047 strain in this pathosystem. WRKY39 has been associated with pathogen defense and JA signaling [55]. WRKY39 enhances resistance to abiotic stress through proline accumulation, as well as to *Pseudomonas syringae* pv. tomato DC30000 (PstDC300) through overexpression of PR1 [56]. Our data did not show PR1 gene expression in priming treatment, indicating that WRKY39 involved other defense mechanisms triggered by BBC047. In addition, the crosstalk regulation physiological process has been described for WRKY transcription factor AtWRKY39 biotic and abiotic stress response [55], whereas in rice, different allele variants of OsWRKY45 can respond to SA and/or JA levels [56].

In N6, the expression of detoxification and cell wall synthesis genes (Supplementary Table S7) was observed. The overexpression of these genes shows that priming by BBC047 induces defense-related genes and activates mechanisms to cope with the damage produced by the pathogen and the oxidative burst activated in the tomato leaf cells. ISR activation is accompanied by HR with accumulation of ROS, which can cause cellular damage, even in the non-infected cells. In the priming treatment, we observed overexpression of two gene models annotated as glutathione S-transferase (GST), an ubiquitous molecule involved in the detoxification and attenuation of oxidative stress activated with the H_2_O_2_ accumulation [57]. It interesting to note that GST has been reported to be involved in resistance gene (R)-mediated defense and with ISR [53], where its increment is highly associated with the gene expression of R genes and with pre-inoculation with beneficial bacteria. Interestingly, it was shown that following pretreatment with tyrosine kinase or with a serine/threonine inhibitor (RLK inhibitors), the oxidative burst and GST expression is suppressed [58]. In this work, overexpression of a subset of RLK genes was also observed in the priming treatment, which might be involved with the upregulation of GST through ROS augmentation, given the signaling pathway activated by the RLKs. These data support the hypothesis that pretreatment with BBC047 triggers ISR in tomato plants against *B. cinerea* infection.

In N6 the oxalate oxidase-like germin (Solyc07g041720.1) was also observed. Previously, this gene was reported as PR10, a part of the plant protein family in the capacity of degradation of oxalic acid (OA), a toxic compound secreted by *B. cinerea* in the early stages of plant infection [59]. However, here, it was over-represented only in the priming treatment. Degradation of OA by this protein produces H_2_O_2_ as a by-product [60], which is known as part of the signaling transduction cascade, resulting in the activation of defense mechanisms, such an as the activation of GST.

### 4.3. Gene Expression in the Curative Treatment with BBC047 Strain

The order in which plant PGPRs and fungus are incorporated in the pathosystem generates contrasting defense responses in tomato plants. Whereas priming treatment induces a pre-conditioning stage that facilitates the ISR response when the *B. cinerea* colonize the plant tissues, the application of BBC047 after *B. cinerea* inoculation (curative treatment) increased gene expression associated with SAR response.

SAR involves different stages: signal generation in primary contact, systemic translocation of the SAR signals (SA, glycerol 3-phosphate (G3P), etc.), signal perception in the systemic tissue and induction of a “defense-ready” status [61]. The curative treatment induced two genes related to G3P: glycerol 3-phosphate transporter and enzyme glycerol 3-phosphate acyltransferase. G3P is a metabolite with a critical role as a mobile inducer of systemic immunity in plants, is accumulated in early stages (6–16 h after infection) and is essential for SAR induction [62,63], which could explain the overexpression of G3P transporter in our results. Other authors have also reported that an intact cuticle is essential for SAR induction. The cuticle is composed of cuticular waxes and cutin monomers, which are derived from FAs synthetized for the enzyme GPAT using G3P as precursor [64]. In addition, AA (azelaic acid) and G3P are described as systemic defense inducers associated with SAR. Yu et al. [65] suggested that the inoculation of a pathogen triggers the release of free, unsaturated C18 FAs, which undergo oxidative cleavage at carbon 9 to serve as AA precursors. Then, AA accumulation activates G3P biosynthesis, and the enzyme glycerol 3-phosphate acyltransferase converts G3P to phosphatidic acid (PA), which could be incorporated in the synthesis of azelaic acid. Four Cyt450 (Solyc03g111995.1, Solyc03g116630.3, Solyc07g055560.3 and Solyc10g083690.3) probably involved on FA oxidation and two other carbon sources for PA synthesis (diacylglycerol (DAG) kinase and phospholipase PLDb2) were identified. PA could be synthetized through the enzymes phosphatidylinositol:ceramide and inositolphosphotransferase and then via the diacylglycerol kinase or the phospholipase D (PLD) pathway [66], a pathway that can be activated through SA and therefore play a role in the early steps of the SA-triggered transduction and activation of an SAR response [67]. Therefore, our data show that the curative treatment could be involved the induction of molecular signals associated with SAR response stronger than in the P + F treatment, suggesting that the application of a beneficial microorganism after fungal infection could extend the synthesis of mobile signals that are generally accumulated in response to infection within the first 4–6 h and intensify the susceptibility to infection by *B. cinerea* in the curative treatment.

The curative treatment also induced modifications in the secondary cell wall, activating the lignin biosynthesis pathway. Previously, Gallego-Giraldo et al. [68] described that plants with similar lignin quantity but different compositions have different transcriptomic profiles during plant defense. Here, distal tissues over-represented two MYB transcription factors, MYB75 (described as LsAN2 in tomato) and MYB58. In *Arabidopsis*, MYB75 has two well-identified functions: promotion of anthocyanin biosynthesis in vegetative tissues and as a negative regulator of the whole secondary cell wall program [69,70]. On the other hand, MYB58 is a positive regulator of the genes involved in lignin biosynthesis and secondary wall formation [71]. Despite the contrary role in the secondary wall formation, both are over-represented in our study, demonstrating crosstalk regulation of these physiological processes.

In addition, enzymes of lignin biosynthesis HCT, C4H, COMT and CCoAOMT were identified in the curative treatment. Gallego-Giraldo et al. [72] reported that multiple PR proteins were expressed in antisense plant HCT, as well an inversely proportional levels of salicylic acid vs. the lignin levels in plants, whereas HCT is downregulated [73]. These findings could explain the absence of PRs in the curative treatment, whereas the application of BBC047 could also reduce the SA levels. Another interesting aspect is the over-representation of C4H and COMT, whereas CCoAOMT is downregulated. COMT and CCoAOMT have similar functions in the lignin metabolic pathway; therefore, this contrary expression could be explained by preference for synthesis of a specific metabolite. Different cell wall compounds are linked to different plant responses to biotic stress conditions [68]. Further research is necessary to determine whether such cell wall modifications improve or are detrimental for response to fungus attacks.

On the other hand, two Jasmonate Zim domain—JAZ genes were found (JAZ3 and JAZ8; Solyc07g042170.3 and Solyc08g036620.3, respectively). JAZ proteins repress the transcription factor MYC2, which regulates different subsets of the JA-dependent transcriptional response [74]. This information is concordant with previous results; the SAR response reflected in our results is probably induced by SA. SA and JA exhibit an opposite response under SAR or ISR response; therefore, in the curative treatment repression of the JA response would enhance the SAR response.

Finally, two chaperons (HSP70 and HSP90) were found in the curative treatment. These proteins were described as essential components of plant defense signaling. When HSP90 were silenced in tobacco plants, the expression of PR1 and other PR genes were consistently reduced, in addition to a hypersensitive response [75]. The chaperon HSP70 was found to enhance effector-induced cell death [76]. In our assay, only the infection control (P + F) showed expression of PR genes, probably due to cross-talk regulation in infection and the curative application of BBC047 in the P + F + B treatment. In addition, Govrin and Levine [77] showed that necrotrophic fungi, such as *B. cinerea*, trigger HR, which results in cell death and facilitates its colonization of plants. Therefore, the overexpression of HSP70 and HSP90, both essential components of the hypersensitive response (HR) defense mechanism [75], could increase the susceptibility to *B. cinerea* under curative treatment conditions.

## 5. Conclusions

**Figure 5:**
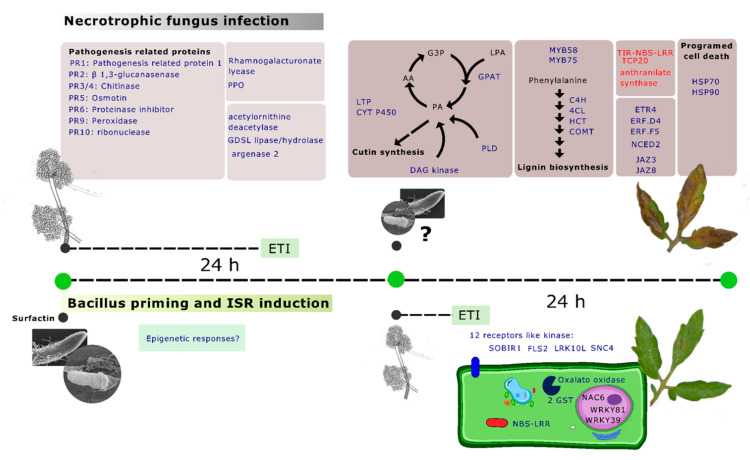
Schematic representation of the “plant–bacteria–fungi” pathosystem evaluated in this study. (**Top**): gene expression profile in P + F and P + F + B at 24 h after *B. cinerea* infection. A set of pathogenesis-related proteins is activated (**left**); however, the incorporation of BBC047 into the system (**right**) generates the worst damage in tissues expressed as genes related to SAR signals, as well as lignin biosynthesis and programed cell death. (**Bottom**): comparison of molecular response in the P + B and P + B + F treatments. Although few genes were differentially expressed between CP and P + B treatments, the incorporation of *B. cinerea* induced receptor-like kinases, NBS-LRR, transcription factors and related genes to antioxidant systems. Blue represents expressed genes, and red represents repressed genes.

In our study, we explored the plant defense response induced by *B. velezensis* BBC047 in relation to its application before or after the *B. cinerea* infection of tomato plants. Figure 5 summarizes our main findings derived from the observed gene expression profiles in the different experimental assemblies of the pathosystem of tomato—*B. cinerea—B. velezensis* strain.

The inoculation of BBC047 did not considerably alter the gene expression of tomato tissues, whereas infection with *B. cinerea* in BBC047-primed plants (P + B + F) induced the expression of LRR and NBS-LRR receptors, which are highly related to the ISR response. On the other hand, in the infected control treatment (P + F), we detected molecular patterns typical of defense response to pathogen infection, mainly based on expression of pathogenesis-related protein (PR) genes in systemic tissue.

Unlike many other studies, our experimental design considered a curative treatment (P + F + B), which allowed us to gain insights into the plant response to inverted priming. In this treatment, *B. cinerea* caused the most tissue damage, extending nearly entirely across the infected leaves. Additionally, genes generally associated with early SAR response (<16 h) were overexpressed, and apparently, the beneficial strain was not perceived as such. Therefore, we infer that the plant defense in the curative treatment represents a higher degree of biological stress triggered by the incorporation of strain BBC047 as a second arriving microorganism. However, further studies are needed on the relevance of the timing of exposure to a beneficial microorganism and a pathogen.

The use of beneficial microorganisms in agricultural systems does not always achieve the desired results, which reduces the willingness of farmers to incorporate such technology in their crop management protocols. Our findings could contribute to offset this situation by providing relevant information on the development and improvement of integrated pest management strategies in sustainable agriculture. As we showed here, the (undetected) presence of pathogen infection in a field and later application of beneficial microbes could increase the biotic stress instead of enhancing plant resistance via ISR. Hence, knowing the phytosanitary status of plants prior to the inoculation of a beneficial microorganism is important for successful ISR induction. Appropriate timing of the inoculation with beneficial microorganisms could prevent greater damage to crops, avoiding accumulation of multiple stresses (transplant, biotic and abiotic stresses). Nonetheless, further research is required, particularly under environmentally variable conditions, as typical for agricultural systems.

## Figures and Tables

**Table 1:** Quantitative data for surfactin production in each of the treatments.

Treatment	Surfactin C Concentration (µg/mL)	SD *n* = 3	Bacterial Cell Count (cfu/mL)	Surfactin C Concentration (µg/mL) Normalized by Bacterial Cell Count
Control (CB)	45.8	1.8	42,520,000	45.8 ± 1.8
P + B	123.4	4.1	9,242,500	567.6 ± 19.0
P + B + F	9.2	4.5	1,275,000	308.1 ± 149.6
P + F + B	15.5	1.2	1,835,000	358.0 ± 26.8

**Table 2:** Pathogenesis-related proteins identified in N3.

ID	Description	Function
Solyc09g007010.1.1	Pathogenesis-related protein 1, PR1	Pathogenesis-related protein 1
Solyc10g079860.2.1	Pathogenesis-related protein 2, PR2	Beta(1,3)glucanase
Solyc10g055810.2.1	Pathogenesis-related protein 3, PR3	Chitinase
Solyc01g097270.3.1	Pathogenesis-related protein 5, PR5	Pathogen-induced protein (pi1)
Solyc08g080640.2.1	Pathogenesis-related protein 5, PR5	Osmotin-like protein (fragment) IPR017949 Thaumatin, conserved site IPR001938
Solyc08g080650.3.1	Pathogenesis-related protein 5, PR5	Osmotin-like protein (Fragment) IPR001938 Thaumatin, pathogenesis-related
Solyc00g145170.2.1	Pathogenesis-related protein 6, PR6	Proteinase inhibitor II
Solyc01g067295.1.1	Pathogenesis-related protein 6, PR6	Metallocarboxypeptidase inhibitor
Solyc03g098710.1.1	Pathogenesis-related protein 6, PR6	Kunitz trypsin inhibitor
Solyc03g098720.3.1	Pathogenesis-related protein 6, PR6	Kunitz trypsin inhibitor
Solyc03g098780.2.1	Pathogenesis-related protein 6, PR6	Cathepsin D Inhibitor
Solyc03g098790.3.1	Pathogenesis-related protein 6, PR6	Cathepsin D Inhibitor
Solyc07g007250.3.1	Pathogenesis-related protein 6, PR6	Metallocarboxypeptidase inhibitor
Solyc07g007260.3.1	Pathogenesis-related protein 6, PR6	Metallocarboxypeptidase inhibitor
Solyc09g083440.3.1	Pathogenesis-related protein 6, PR6	PIN-I protein
Solyc09g084450.3.1	Pathogenesis-related protein 6, PR6	Proteinase inhibitor I
Solyc09g084460.3.1	Pathogenesis-related protein 6, PR6	Proteinase inhibitor I
Solyc09g084465.1.1	Pathogenesis-related protein 6, PR6	Wound-induced proteinase inhibitor 1
Solyc09g089500.3.1	Pathogenesis-related protein 6, PR6	Proteinase inhibitor I
Solyc09g089510.3.1	Pathogenesis-related protein 6, PR6	Proteinase inhibitor I
Solyc09g089530.3.1	Pathogenesis-related protein 6, PR6	PIN-I protein
Solyc09g089540.3.1	Pathogenesis-related protein 6, PR6	Proteinase inhibitor I
Solyc11g021060.2.1	Pathogenesis-related protein 6, PR6	TOMARPIX proteinase inhibitor
Solyc11g022590.1.1	Pathogenesis-related protein 6, PR6	Trypsin inhibitor-like protein precursor
Solyc00g071180.3.1	Pathogenesis-related protein 6, PR6	Multicystatin-cysteine protease inhibitor
Solyc09g089500.3.1	Pathogenesis-related protein 6, PR6	Proteinase inhibitor I (AHRD V3.3 K7WNW8_SOLTU)
Solyc09g089510.3.1	Pathogenesis-related protein 6, PR6	Proteinase inhibitor I (AHRD V3.3 K7WNW8_SOLTU)
Solyc01g067295.1.1	Pathogenesis-related protein 6, PR6	Metallocarboxypeptidase inhibitor (AHRD V3.3 O24373_SOLTU)
Solyc01g006290.3.1	Pathogenesis-related protein 9, PR9	Peroxidase
Solyc07g006560.3.1	Pathogenesis-related protein 10, PR10	Hypersensitive response assisting protein
Solyc07g006570.3.1	Pathogenesis-related protein 10, PR10	Ribonuclease 3

**Table 3:** Receptors identified in response to priming treatment (N6).

ID	Description	Domain	Location
Leucine-rich receptor-like serine/threonine protein kinase			
Solyc02g072470.3.1	GSO1	Cytoplasmic serine/threonine kinase domain Extracellular leucine-rich repeat domain	Plasma membrane
Solyc03g111793.1.1	Suppressor of BIR1 1, SOBIR1	Cytoplasmic serine/threonine kinase domain Extracellular leucine-rich repeat domain	Plasma membrane
Solyc04g012100.2.1	Lipase of Fusarium solani 2, FSL2	Cytoplasmic serine/threonine kinase domain Extracellular leucine-rich repeat domain	Plasma membrane
Receptor-like kinases containing leucine-rich repeats (LRRs)			
Solyc04g074000.3.1	MDIS1-interacting receptor like kinase 2, MIK2	Extracellular leucine-rich repeat domain Cytoplasmic kinase domain	Plasma membrane
Solyc04g074030.3.1	MDIS1-interacting receptor like kinase 2, MIK2	Extracellular leucine-rich repeat domain Cytoplasmic kinase domain	Plasma membrane
Solyc04g074050.3.1	MDIS1-interacting receptor like kinase 2, MIK2	Extracellular leucine-rich repeat domain Cytoplasmic kinase domain	Plasma membrane
Solyc05g008950.3.1	MDIS1-interacting receptor like kinase 2, MIK2	Extracellular leucine-rich repeat domain Cytoplasmic kinase domain	Plasma membrane
Solyc05g008960.3.1	Leaf rust 10 disease-resistance locus receptor-like protein kinase 4, LRK10L4	Cytoplasmic serine/threonine kinase domain Extracellular leucine-rich repeat domain	Plasma membrane
Solyc06g048740.2.1	Probable LRR receptor-like kinase	Cytoplasmic aerine/threonine kinase domain Extracellular leucine-rich repeat domain	Plasma membrane
Solyc07g055810.3.1	Probable LRR receptor-like kinase	Cytoplasmic serine/threonine kinase domain Extracellular leucine-rich repeat domain	Plasma membrane
Receptor-like protein kinase			
Solyc05g009040.3.1	Suppressor of npr1-1—constitutive 4, SNC4	Receptor-like kinase with two extracellular glycerophosphoryl diester phosphodiesterase domains	Plasma membrane
Solyc10g076550.1.1	Receptor-like protein kinase. WAK1	EGF-like domain	Plasma membrane
Solyc09g083200.3.1	Nod factor receptor protein (LYK4)	Lys motif	Plasma membrane
Disease-resistance protein, NBS-LRR class family			
Solyc04g007070.3.1	R gene—RPP 13	NBS-LRR class family	Cytoplasm

## Data Availability

The data presented in this study are included this article and its supplements. Original data from RNASeq are available upon request to the corresponding authors.

## Supplementary Materials

The following supporting information can be downloaded at: https://www.mdpi.com/article/10.3390/microorganisms10081547/s1, Figure S1: Calibration curve for surfactin C based on surfactin standard (CAS N° 24730-31-2, Sigma Aldrich, Germany) generated by injecting four known surfactin concentrations (2.5, 25, 50 and 100 μg/mL); Figure S2: Schematic representation of the inoculation/infection and sampling strategy in order to obtain tomato leaflets for analysis of systemic defense response via RNASeq.; Figure S3: Effect of the different pathosystem Plant—BBC047- B. cinerea assemblies on disease severity of B. cinerea. Disease reduction index and lesion area are shown; Figure S4: Mass spectrum for cLP per treatment; Figure S5: Gene expression comparison between RNAseq data and qPCR analysis. The genes ERF.F5, HSP, JAZ8, JAZ3, NBS-LRR, Anexin 5, MYB58 and MYB75 were evaluated. Bars represent error bars. A Tukey test was performed has a post hoc comparison; Table S1: qPCR primers list; Table S2: Summary of sequenced libraries for each plant treatment after filtering and genome mapping; Table S3: Summary of gene expression levels of five plant treatments; Table S4: Differential gene expression analysis. For comparison, first read column 1 and then row 2. Top and bottom sections refer to induced and repressed genes, respectively; Table S5: Gene composition of each node obtained from SOM analysis using differentially expressed genes. XLOC and Solyc Ids correspond to transcript codes obtained from RNAseq analysis and tomato genome project, respectively; Table S6: N3-expressed genes without pathogenesis-related proteins; Table S7: N6-expressed genes and PRR-related proteins; Table S8: Differentially expressed genes identified as overexpressed (Node 2) and repressed (Node 4) in curative treatment.
