# Supplementary material for: New Insights into Bacillus-Primed Plant Responses to a Necrotrophic Pathogen Derived from the Tomato-Botrytis Pathosystem
Source: Microorganisms. 2022 Jul 30;10(8):1547. doi: 10.3390/microorganisms10081547 (PMC9416759; doi:10.3390/microorganisms10081547)
Supplement: Supplementary file 1 [file microorganisms-10-01547-s001.zip › Supplementary Figures_12_07_2022.pdf]

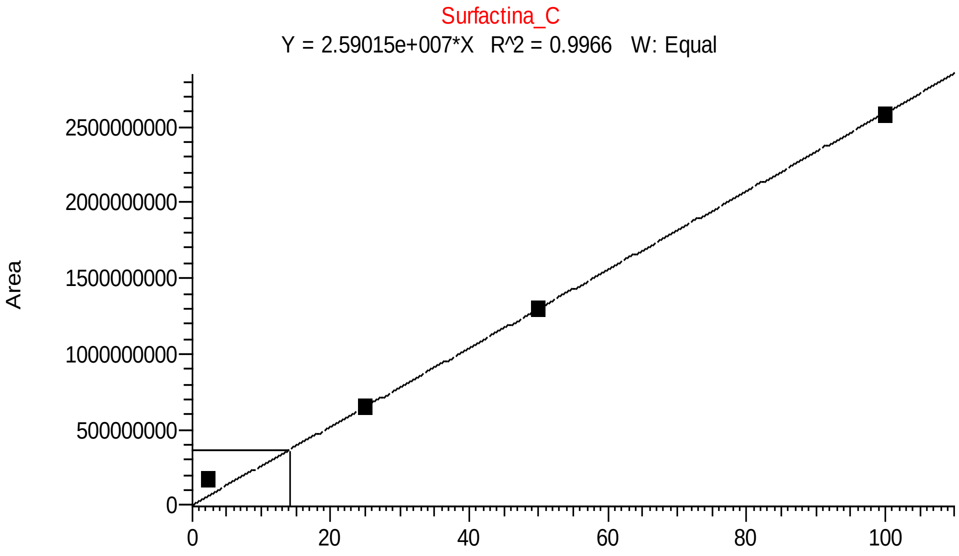

Supplementary Figure S1. Calibration curve for surfactin C based on surfactin standard (CAS N° 24730-31-2, Sigma Aldrich, Germany), generated by injecting 4 known surfactin concentration (2.5, 25, 50 and 100 µg/mL)

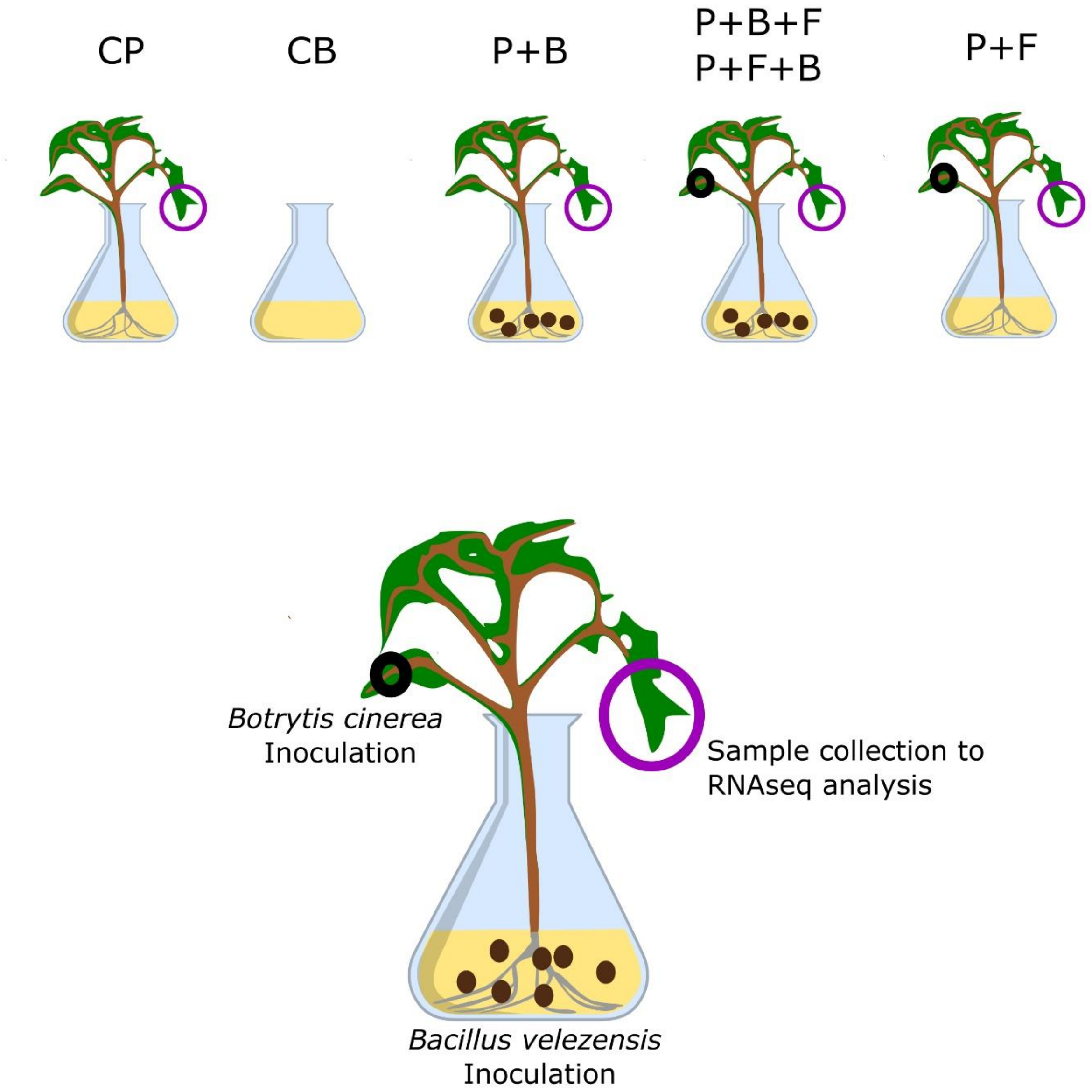

Supplementary Figure S2. Schematic representation of the inoculation/infection and sampling strategy in order to obtain tomato leaflets for the analysis of systemic defense response via RNASeq. Please note that the inoculated (root)/infected (one pair of leaflet) tissue is distant from the sampled tissue for RNASeq analysis (another pair of leafleats).

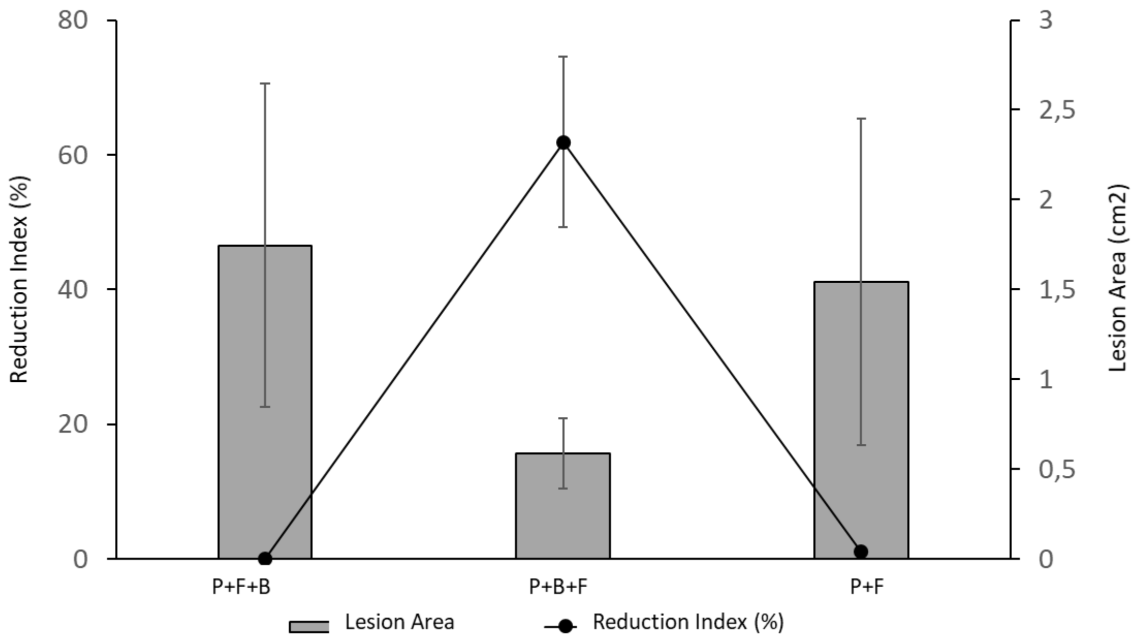

Supplementary Figure S3. Effect of the different pathosystem Plant - BBC047- *B. cinerea* assemblies on the disease severity of *B. cinerea*. Disease Reduction Index and Lesion area are shown.

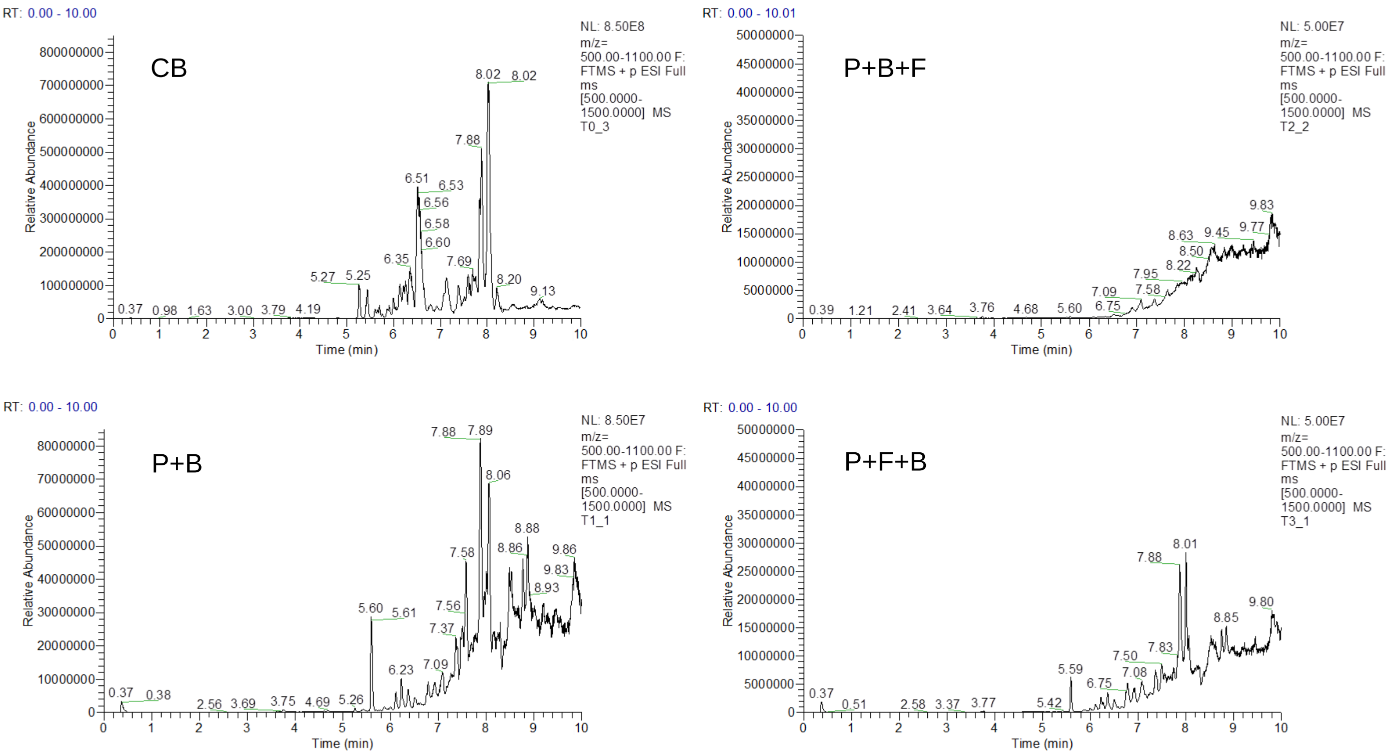

Supplementary Figure S4. Mass spectrum for cLP per treatment, <u>note:</u> y-axis of the graphs was adjusted to improve visualization of each curve.

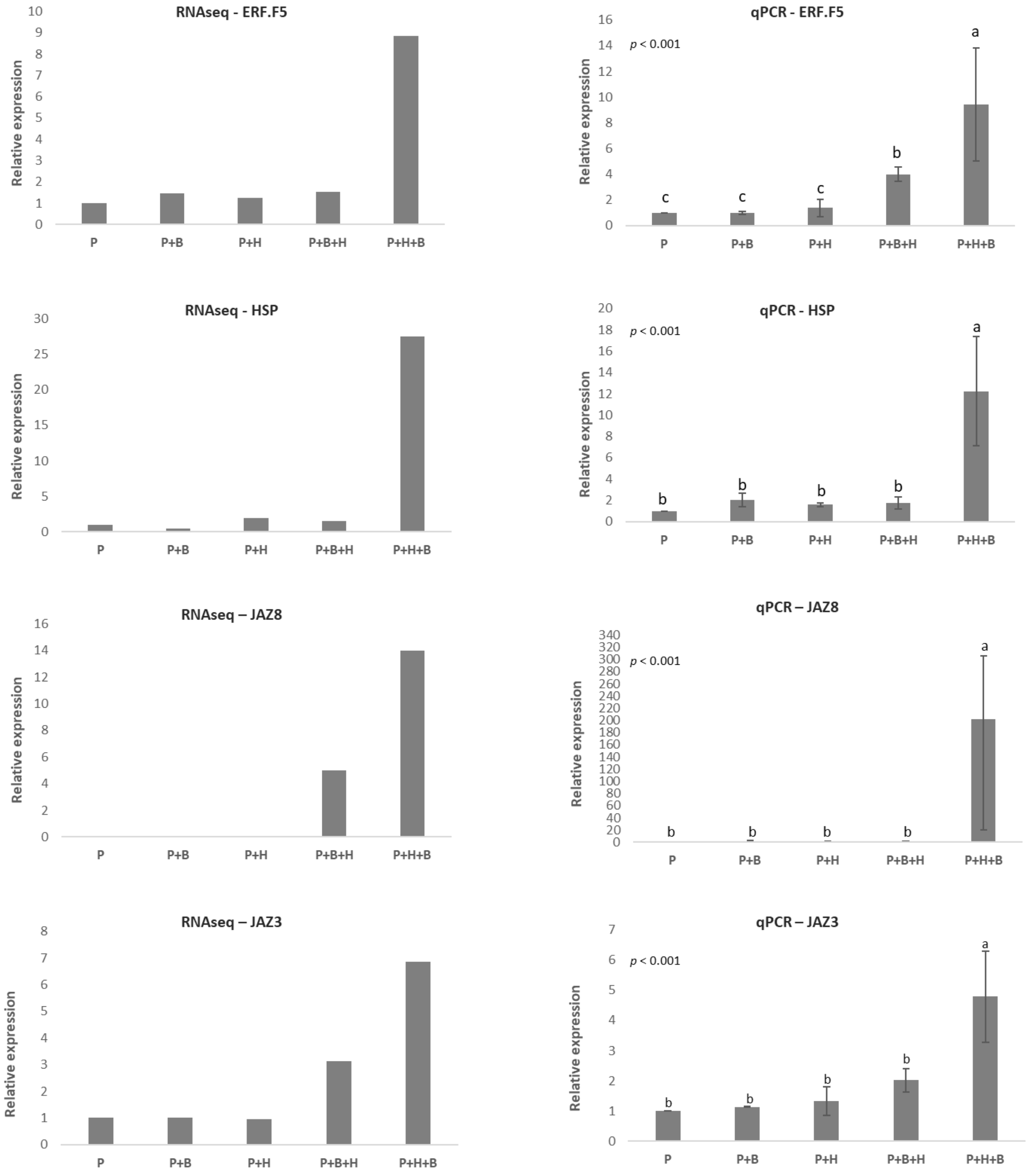

Supplementary Figure S5. Gene expression comparison between RNAseq data and qPCR analysis. The genes ERF.F5, HSP, JAZ8, JAZ3, NBS-LRR, Anexin 5, MYB58 y MYB75 were evaluated. Bars represent Error Bars. Tukey test was performed has post-doc comparison.

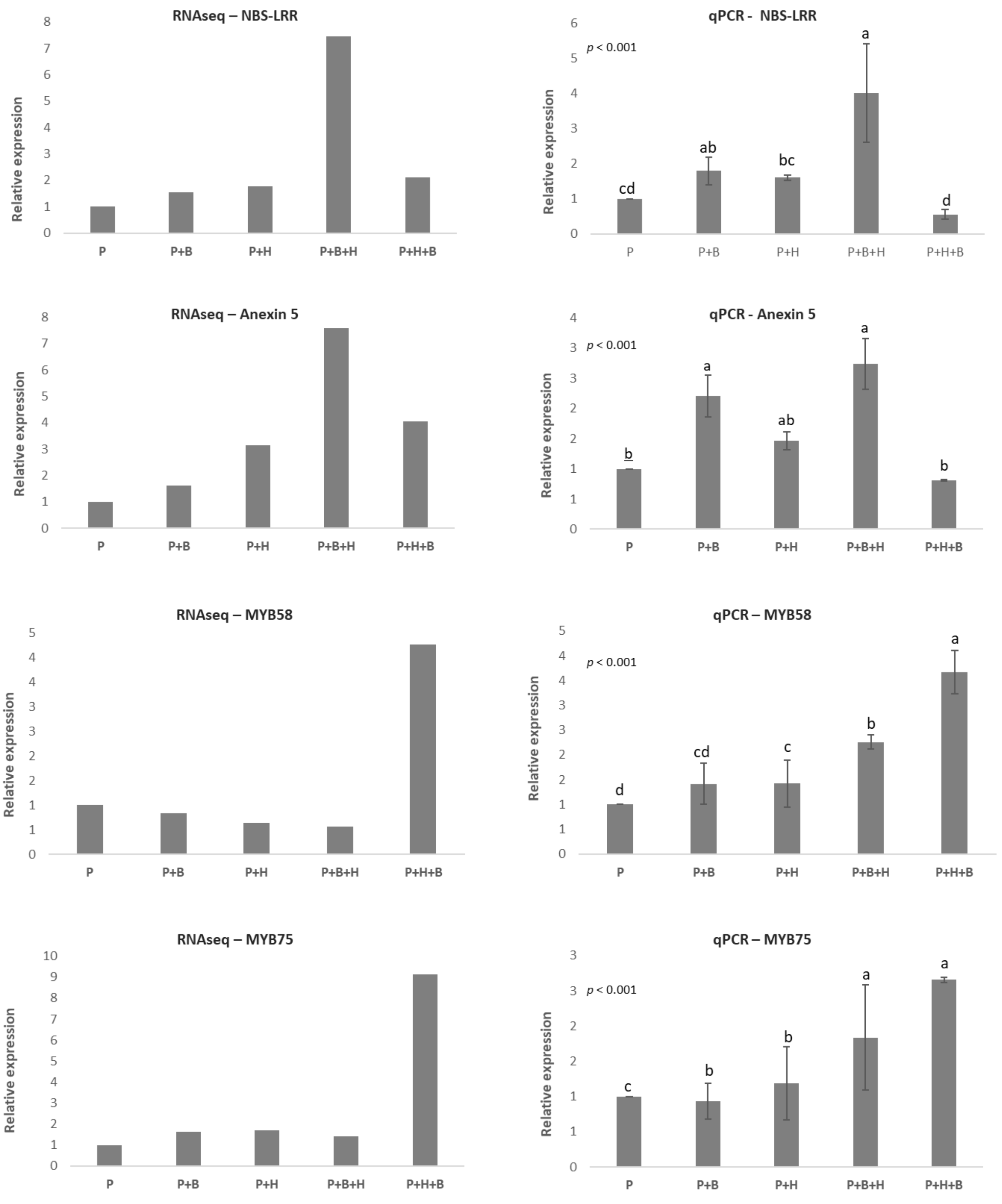

Supplementary Figure S5 (continued). Gene expression comparison between RNAseq data and qPCR analysis. The genes ERF.F5, HSP, JAZ8, JAZ3, NBS-LRR, Anexin 5, MYB58 y MYB75 were evaluated. Bars represent Error Bars. Tukey test was performed has post-doc comparison.
